# Platelet-Rich Plasma Therapy for Rotator Cuff Injuries: A Comprehensive Review of Current Evidence and Future Directions

**DOI:** 10.7759/cureus.70042

**Published:** 2024-09-23

**Authors:** Vinit Rathod, Sandeep Shrivastav, Milind R Gharpinde

**Affiliations:** 1 Orthopaedics, Jawaharlal Nehru Medical College, Datta Meghe Institute of Higher Education & Research, Wardha, IND

**Keywords:** clinical trials, growth factors, platelet-rich plasma (prp), rotator cuff injuries, shoulder pain, tendon repair

## Abstract

Rotator cuff injuries are a prevalent cause of shoulder pain and disability, significantly impacting daily activities and quality of life. Platelet-rich plasma (PRP) therapy has emerged as a potential treatment for these injuries, aiming to enhance healing by delivering concentrated platelets and growth factors. This review comprehensively evaluates the current evidence regarding PRP therapy for rotator cuff injuries. It examines clinical trial data, comparing PRP therapy with conventional treatments such as physical therapy and surgical intervention. The review also explores the biological mechanisms of PRP, including its role in promoting tendon repair and regeneration through growth factors and cytokines. In addition, it addresses variables that may affect PRP therapy outcomes, including preparation techniques, injection methods, and patient-specific factors. The review highlights the need for standardized protocols and further research to optimize PRP therapy and address existing gaps in knowledge. Future directions include exploring combined treatment approaches and assessing long-term outcomes to refine PRP therapy's role in rotator cuff injury management. This review aims to provide valuable insights into the effectiveness of PRP therapy, contributing to improved treatment strategies and enhanced patient outcomes.

## Introduction and background

Rotator cuff injuries are a common and significant cause of shoulder pain and disability, affecting the group of muscles and tendons responsible for stabilizing and moving the shoulder joint [1]. The rotator cuff comprises four key muscles: the supraspinatus, infraspinatus, teres minor, and subscapularis. These muscles and their associated tendons play a crucial role in shoulder stability and movement. Injuries to the rotator cuff can range from tendinitis, characterized by inflammation of the tendons, to tendinosis, which involves degenerative changes without inflammation [2]. More severe cases involve rotator cuff tears, which can be partial or full-thickness. Partial tears damage a portion of the tendon, while full-thickness tears result in a complete rupture. These injuries can occur acutely, such as from trauma, or gradually, due to repetitive stress and degeneration over time [1].

The epidemiology of rotator cuff injuries indicates that they are particularly prevalent among middle-aged and older adults, with an increased incidence in individuals engaged in repetitive overhead activities or heavy lifting [3]. The impact of these injuries on quality of life is substantial, as they often lead to pain, limited shoulder mobility, and functional impairment. This can significantly affect an individual’s ability to perform everyday tasks and participate in recreational activities. The chronic nature of rotator cuff injuries can lead to long-term disability and decreased quality of life if not appropriately managed [4]. Platelet-rich plasma (PRP) therapy has emerged as a potential treatment option for enhancing the healing of rotator cuff injuries. PRP therapy involves using a concentrated preparation of platelets derived from the patient’s blood. To prepare PRP, a blood sample is centrifuged to concentrate the platelets rich in growth factors and cytokines. This concentrated PRP is then injected into the injury site to accelerate the body’s natural healing processes [5].

The mechanism of action of PRP therapy is based on its ability to deliver a high concentration of growth factors directly to the injury site. These growth factors play a crucial role in promoting cellular proliferation, collagen synthesis, and angiogenesis, which are essential for the repair and regeneration of tendons. By creating an optimal healing environment, PRP therapy aims to enhance tendon repair, reduce inflammation, and alleviate pain [6]. This review aims to provide a comprehensive evaluation of the current evidence regarding the efficacy of PRP therapy in treating rotator cuff injuries. This review will examine the available clinical trial data, discuss the underlying biological mechanisms of PRP therapy, and explore various factors that may influence treatment outcomes. In addition, it will highlight potential future research directions and improvements in PRP therapy to provide a thorough understanding of its role in managing rotator cuff injuries.

## Review

Current evidence on PRP therapy for rotator cuff injuries

Current evidence on PRP therapy for rotator cuff injuries underscores its efficacy, safety, and comparative performance against conventional treatments. This review consolidates key clinical trials, methodologies, efficacy outcomes, and safety considerations [7]. The studies generally involved randomized controlled trials with sample sizes ranging from 30 to 70 participants. Common methodologies included evaluating pain levels, range of motion (ROM), and functional scores at baseline, post-treatment, and follow-up intervals (e.g., six weeks and 12 months) [8]. One study demonstrated that ultrasound-guided PRP injections significantly improved pain (measured by the Visual Analog Scale), shoulder ROM, and function (assessed by the DASH score) compared to physical therapy in patients with chronic partial supraspinatus tears [9]. Another study involving 70 patients with chronic partial supraspinatus tears compared PRP injections with physical therapy and found significant improvements in both groups over 12 months. However, the PRP group achieved better DASH scores, indicating superior functional outcomes despite no significant differences in pain reduction or ROM compared to the physical therapy group [10]. PRP and physical therapy resulted in significant pain reduction, but PRP often showed superior outcomes in functional improvement as measured by DASH scores. PRP injections led to better functional results than physical therapy alone, although both approaches demonstrated progress over time [11]. PRP injections have been shown to offer enhanced outcomes in terms of pain relief and functional improvement, particularly in chronic cases. However, physical therapy remains an essential component of rehabilitation and can be effectively combined with PRP for improved results [12]. While PRP presents a promising alternative to surgery, it may not fully replace surgical options for severe rotator cuff injuries. Instead, PRP may be an interim solution or a conservative management strategy for partial tears before surgery. PRP therapy is generally well-tolerated, with common side effects including localized pain at the injection site, swelling, and bruising, which are typically mild and transient [13]. Serious complications are rare but can include infection, nerve damage, or adverse reactions to the anesthetic used during the procedure. Overall, the safety profile of PRP is favorable, especially when compared to more invasive treatments like surgery [14].

Mechanisms of action

PRP therapy is based on the biological properties of platelets, which are essential not only for blood clotting but also for tissue healing and regeneration. PRP is rich in growth factors and cytokines that are crucial for directing the healing process [15]. Key components include platelet-derived growth factor (PDGF), which promotes cell proliferation and angiogenesis, thus facilitating healing. Transforming growth factor-beta (TGF-β) contributes to collagen synthesis and tissue remodeling [16]. In addition, vascular endothelial growth factor (VEGF) stimulates the formation of new blood vessels, improving blood supply to the injured area, while epidermal growth factor (EGF) supports cell growth and differentiation. These growth factors are released upon platelet activation and play essential roles in attracting reparative cells to the injury site, thereby supporting the healing cascade [16]. Platelets play a multifaceted role in healing beyond just releasing growth factors. They stimulate cell proliferation, particularly of mesenchymal stem cells (MSCs) and other reparative cells crucial for tissue regeneration. Platelets also help modulate the inflammatory response by releasing anti-inflammatory cytokines, vital for initiating the healing process and reducing pain associated with injuries. This comprehensive role of platelets highlights the biological basis of PRP therapy and its potential effectiveness in treating various musculoskeletal injuries, including rotator cuff injuries [17]. PRP therapy has significantly affected tendon repair and regeneration in the context of rotator cuff injuries. Applying PRP directly to the injury site enhances collagen synthesis, vital for maintaining tendon strength and integrity [18]. The growth factors in PRP stimulate collagen production, promoting the structural restoration of the tendon. In addition, PRP supports angiogenesis, mainly through VEGF, which aids in forming new blood vessels. Improved vascularization enhances nutrient delivery and waste removal in the healing tissue, further supporting recovery [19]. PRP therapy also plays a crucial role in managing inflammation and pain associated with rotator cuff injuries. By reducing pro-inflammatory cytokines such as IL-1β, PRP effectively alleviates inflammation at the injury site. This modulation of the inflammatory response promotes healing and reduces pain, leading to improved patient outcomes and functional recovery [20]. The combination of enhanced tissue regeneration and reduced inflammation positions PRP therapy as a promising treatment option for individuals with rotator cuff injuries, underscoring its potential to facilitate a more effective and efficient healing process [21]. Detailed mechanisms of PRP therapy in rotator cuff injury repair are outlined in Table 1.

**Table 1:** Detailed mechanisms of the action of platelet-rich plasma (PRP) therapy in rotator cuff injury repair

Mechanism	Detailed description
Growth factor release [15]	PRP contains high concentrations of growth factors such as Platelet-Derived Growth Factor (PDGF), Vascular Endothelial Growth Factor (VEGF), and Transforming Growth Factor-Beta (TGF-β). These factors stimulate cell proliferation, tissue repair, and angiogenesis. PDGF promotes the recruitment of mesenchymal stem cells and fibroblasts, essential for tendon repair. VEGF enhances vascularization, which is critical for delivering oxygen and nutrients to the damaged tissue, while TGF-β helps regulate inflammation and tissue remodeling.
Angiogenesis stimulation [22]	PRP therapy boosts the formation of new blood vessels (angiogenesis) by upregulating angiogenic factors like VEGF. Increased vascularization ensures enhanced oxygen and nutrient supply to the damaged rotator cuff tendon, accelerating healing. This mechanism is particularly beneficial in tendons, which naturally have a poor blood supply, helping to nourish the injury site and create an optimal environment for repair.
Collagen synthesis [23]	Collagen is a primary structural component of tendons. PRP promotes the activation of fibroblasts, which play a critical role in producing collagen. The increased collagen synthesis reinforces the extracellular matrix, aiding in tendon strength and flexibility. This process is vital for restoring the structural integrity of the damaged rotator cuff, leading to a stronger and more resilient tendon.
Anti-inflammatory effect [24]	One of PRP's significant benefits is its ability to modulate inflammation. PRP contains molecules like interleukin-1 receptor antagonists (IL-1Ra) that reduce pro-inflammatory cytokines like IL-1 and TNF-α. By controlling the inflammatory response, PRP therapy minimizes pain and swelling, creating a conducive environment for tissue regeneration. This anti-inflammatory action is crucial for limiting chronic inflammation, which can impede the healing process in rotator cuff injuries.
Cellular migration [25]	PRP attracts various types of cells, such as stem cells, fibroblasts, and tenocytes, to the injury site by releasing chemotactic agents. These cells are essential for tissue regeneration and play a role in rebuilding damaged tendon tissue. The migration of cells to the site of injury is crucial for initiating the repair process, particularly in the context of tendon healing, where cellular activity can be limited due to poor blood supply.
Tissue remodeling [26]	PRP not only promotes initial tissue repair but also facilitates long-term tissue remodeling. It helps organize the collagen fibers in the tendon, leading to a more structured and functional extracellular matrix. PRP also reduces scar tissue formation by promoting a more balanced and organized tissue repair process. This remodeling phase is essential for restoring normal tendon function and reducing re-injury risk.
Improved tendon-bone healing [27]	Rotator cuff injuries often involve damage to the tendon and the tendon-bone interface. PRP enhances tendon-to-bone healing by promoting fibrocartilage formation and improving the biomechanical properties of the repaired tissue. It supports better integration of the tendon to the bone, strengthening the repair and reducing the likelihood of re-tear. This mechanism is particularly beneficial in surgical repair cases, as it can improve post-surgical outcomes and recovery.

Variables affecting PRP therapy outcomes

Several factors, including preparation techniques, injection methods, and patient-specific variables, can influence the outcomes of PRP therapy. Each of these elements is crucial in determining the efficacy of the treatment for rotator cuff injuries [28]. PRP preparation involves various techniques that can significantly impact its composition and effectiveness. One of the most common methods is centrifugation, where variations in speed and duration can produce different concentrations of platelets and growth factors [29]. Higher centrifugation speeds may increase platelet concentration but could compromise the PRP quality by altering the bioactivity of the growth factors. In addition, the choice of activation methods, such as calcium chloride or thrombin, can affect the release of growth factors. The activation method can influence therapeutic outcomes by modifying the timing and quantity of growth factor release at the injury site [30]. Studies indicate that differences in platelet concentration can result in over tenfold variations in efficacy, impacting pain relief and tissue healing. Therefore, consistency in preparation techniques is essential for achieving reliable clinical results [30]. Injecting PRP into the affected area is also critical to treatment outcomes. Methods such as ultrasound guidance can improve injection accuracy, ensuring that PRP is delivered precisely to the target area, which may enhance clinical results [31]. Administering PRP at multiple sites around the injury may increase the local concentration of growth factors, potentially improving the therapeutic effect. Research suggests that precise targeting of the affected tissue can lead to better pain relief and functional improvement. At the same time, poorly executed injections may result in suboptimal outcomes, highlighting the importance of skilled administration [31]. Patient-specific factors play a significant role in the effectiveness of PRP therapy. Age is a key variable; younger patients may respond more favorably to PRP treatment due to better healing capacities than older individuals, whose regenerative abilities may be reduced [32]. The severity of the injury also influences outcomes; patients with mild to moderate injuries generally experience better results than those with severe or chronic conditions. In addition, comorbidities such as diabetes, obesity, or autoimmune disorders can negatively impact healing and may diminish the effectiveness of PRP therapy. These factors can affect the body’s inflammatory response and tissue regeneration capabilities, ultimately influencing treatment outcomes [33]. Key variables affecting the outcomes of PRP therapy in rotator cuff injuries are summarized in Table 2.

**Table 2:** Key variables affecting the outcomes of platelet-rich plasma (PRP) therapy in rotator cuff injuries

Variable	Description
Platelet concentration [34]	The concentration of platelets in PRP can vary. Higher platelet counts may improve healing by delivering more growth factors, but excessive concentrations could have a diminishing return effect.
Preparation technique [29]	Different centrifugation methods and protocols affect the final PRP composition. Variations in the number of spins, speed, and duration can influence the amount of growth factors and white blood cells in the final product.
Activation method [35]	PRP can be activated before or after injection using agents such as calcium chloride or thrombin. The timing and method of activation affect the release of growth factors and the overall efficacy of the therapy.
Injury severity [36]	The degree of rotator cuff damage (e.g., partial tears vs. full-thickness tears) plays a significant role in determining the effectiveness of PRP. Severe injuries may require additional treatments or longer recovery times.
Patient age [32]	Older patients often have a slower healing process due to reduced regenerative capacity and age-related changes in tissue, which may reduce the efficacy of PRP therapy. Younger patients may experience faster recovery.
Overall health condition [37]	Patients with comorbidities such as diabetes, chronic inflammatory diseases, or obesity may experience slower or reduced healing from PRP therapy due to impaired tissue regeneration and inflammation regulation.
Rehabilitation protocol [38]	Post-injection rehabilitation, including physiotherapy and rest, significantly affects outcomes. Adherence to a tailored rehabilitation plan can enhance PRP therapy's effectiveness in healing rotator cuff injuries.
Injection technique [31]	The accuracy of PRP injection into the damaged tissue (e.g., tendon, tendon-bone interface) is crucial. Ultrasound-guided injections improve precision, ensuring PRP is delivered directly to the injury site.
Number of PRP injections [39]	Multiple PRP injections may yield better results than a single injection, depending on the injury’s severity and the individual’s response to treatment. The timing between injections can also impact outcomes.
Type of PRP used [40]	PRP formulations vary between leukocyte-rich and leukocyte-poor PRP. Leukocyte-rich PRP contains white blood cells, which can enhance or inhibit healing depending on the injury and inflammatory response.

Future directions and research

Innovations in PRP therapy are continually advancing, focusing on improving preparation methods, exploring synergistic effects with other treatments, and understanding long-term outcomes. This review highlights future directions and research needs [17]. Recent studies have introduced novel PRP preparation methods to enhance efficacy and reduce costs. For example, a new cost-effective approach utilizes common laboratory equipment to produce PRP without expensive kits. This method has shown high levels of platelet-derived growth factor (PDGF-BB), especially when using anticoagulants like ACD-A and incorporating platelet aggregation inhibitors such as prostaglandin E1 (PGE1) [41]. In addition, temperature-controlled PRP (t-PRP) preparation has shown promise by avoiding exogenous additives, maintaining physiological characteristics, and promoting better growth factor release and wound healing than conventional methods [42]. Innovations include optimizing centrifugation protocols and preconditioning techniques to enhance PRP's regenerative properties. Continued refinement of PRP preparation techniques is expected to improve the biological activity of the final product. Research indicates that platelet concentration, leukocyte presence, and preparation methods significantly influence PRP's therapeutic effects. Standardization in these areas is crucial for maximizing efficacy and ensuring consistent clinical outcomes [42]. Combining PRP with adjunct therapies, such as physical therapy, has demonstrated potential for improved healing outcomes. Research is investigating how PRP can synergize with rehabilitation protocols to enhance recovery times and functional restoration in patients with rotator cuff injuries and other musculoskeletal conditions [43]. Studies are also exploring the effects of combining PRP with treatments like corticosteroids, hyaluronic acid, and non-steroidal anti-inflammatory drugs (NSAIDs). Understanding the interactions between these therapies and PRP is essential for optimizing treatment protocols and improving patient outcomes [43]. Long-term studies are essential for assessing the durability of PRP's effects on healing and functional improvement. Current literature often lacks sufficient follow-up data, which is necessary to establish the sustained benefits of PRP therapy over time. Future research should focus on longitudinal studies to provide insights into PRP treatments' long-term efficacy and safety. Identifying gaps in current research is crucial for guiding future investigations [44]. Key areas for future research include standardizing PRP preparation methods, establishing uniform protocols to minimize variability in PRP formulations, and improving comparability across studies. In addition, mechanistic studies are needed to elucidate the biological mechanisms underlying PRP's effects, particularly its interactions with immune cells and the inflammatory response. Well-designed, randomized controlled trials are also necessary to validate the effectiveness of PRP in various clinical settings and to compare it with other treatment modalities [45]. Future directions and research priorities in PRP therapy for rotator cuff injuries are summarized in Table 3.

**Table 3:** Future directions and research priorities in platelet-rich plasma (PRP) therapy for rotator cuff injuries

Area of research	Description
Standardization of PRP protocols [29]	Ongoing research aims to establish standardized protocols for PRP preparation, including platelet concentration, activation methods, and injection techniques. This will help reduce variability and improve consistency in outcomes.
PRP in combination with other therapies [46]	Investigating the synergistic effects of PRP combined with other treatments, such as stem cell therapy, hyaluronic acid injections, or corticosteroids, to enhance healing and reduce recovery times in rotator cuff injuries.
Optimizing dosage and injection frequency [47]	Future studies are needed to determine the optimal number of injections, frequency, and timing for PRP therapy. This includes exploring the potential benefits of repeated or booster PRP injections for sustained healing.
PRP for different injury stages [48]	Research is focusing on the efficacy of PRP in treating different stages of rotator cuff injuries, from early-stage tendinopathy to advanced, full-thickness tears, and its potential role in post-surgical recovery.
Long-term outcomes of PRP therapy [49]	More longitudinal studies are required to assess the long-term benefits and durability of PRP therapy for rotator cuff injuries, including patient outcomes, functional improvements, and injury recurrence rates.
Patient-specific PRP formulations [50]	Exploring individualized PRP formulations based on patient characteristics (e.g., age, health conditions, injury severity) to enhance therapeutic outcomes and promote more personalized treatment approaches.
PRP and surgical integration [51]	Investigating how PRP can be used with rotator cuff surgery, either pre-operatively or post-operatively, to improve tissue healing, reduce inflammation, and enhance tendon-bone integration.
Mechanisms of PRP at the molecular level [52]	Future research aims to better understand the precise molecular mechanisms of PRP, including the roles of specific growth factors, cytokines, and cellular interactions that mediate tissue repair and regeneration.
Comparative studies with other treatments [53]	Conducting comparative studies between PRP and conventional treatments (e.g., physical therapy, surgery, corticosteroids) to determine the most effective treatment protocols for different rotator cuff injuries.
Cost-effectiveness of PRP therapy [54]	Evaluating the cost-effectiveness of PRP therapy compared to traditional treatments, particularly in reducing surgery rates, enhancing recovery, and improving long-term patient outcomes for rotator cuff injuries.

## Conclusions

PRP therapy is promising for treating rotator cuff injuries, offering potential benefits in accelerating healing, reducing pain, and improving functional outcomes. The evidence from clinical trials suggests that PRP therapy may be an effective adjunct or alternative to traditional treatments such as physical therapy and surgery, particularly for individuals seeking to avoid more invasive procedures. However, variability in study results highlights the need for standardized protocols and further research to better understand the optimal preparation and application methods. The biological mechanisms underlying PRP therapy, involving growth factors and cytokines, underscore its potential to enhance tendon repair and regeneration. As the field evolves, ongoing research is essential to refine PRP therapy techniques, explore combinatory approaches, and evaluate long-term outcomes. Ultimately, a deeper understanding of PRP therapy's role in rotator cuff injury management will contribute to more personalized and effective treatment strategies, potentially improving the quality of life for individuals affected by these debilitating conditions.
